# A case study of NeuralProphet and nonlinear evaluation for high accuracy prediction in short-term forecasting in PV solar plant

**DOI:** 10.1016/j.heliyon.2022.e10639

**Published:** 2022-09-16

**Authors:** Ricardo Manuel Arias Velásquez

**Affiliations:** Universidad Privada Peruano Alemana, Lima, Perú

**Keywords:** Forecasting, Prediction accuracy, LSTM, NeuralProphet, Convolutional neural network

## Abstract

Prediction of the energy, active production from the PV solar plant is a challenge in cloudy weather or with clouds over the solar plant; therefore, it has impact in the planning of the power system, especially in the season analysis and prediction accuracy adjustments, for example in holidays. In 2022, some authors published some analysis associated to horizontal pyranometers and the limits in the evaluation of the data, the Mean Bias Error of Daily Solar Irradiation average (MIEave) ranges from 0.17% to 2.86% associated a sudden change in the weather, it increases the “risk of misestimating the potential electricity generation” with short-term error of more than 50% and the Global Horizontal Irradiance (GHI) has a mean bias error (MBE) of at least ±8% [1]. In this research article, a novel proposal for short-term forecasting combines the satellite with meteorological station data and statistical model associated to the new seasonality analysis by using two approaches: i) NeuralProphet, Ridge regression, ii) Long Short-Term Memory with convolutional neural networks. Besides, it requires three KPI as feedback, it is the mean absolute error (MAE), relative Root mean square error (RMSE), and mean absolute percentage error (MAPE). The results demonstrate a MAPE of 5.93% and a computational time 852.10 s and the comparison with new predictions methods from 2019 to 2021. This research article illustrates the new approach with the forecasting method in a case of the PV solar plant in Peru and proves the robustness and seasonality results, and new short-terms improvements associated to external influence as cloudy conditions and resource availability. Our findings are an improvement of the model MAPE 12.14%–5.93%; even compared with the literature and currently models as ARIMA-LSTM with 10.57%, LSTM with NN and G, SARIMA and SVM considering Gaussian White Noise with 8.14% and Prophet with SVM with 8.81%.

## 1 Introduction

In the photovoltaic solar plants, the most important external influence is the clouds over the panels; and it is a challenge for the new technologies in the forecasting, especially in tropical conditions.

As well as to reduce the error, several authors provided new solution based on three methods in the last years: i) Satellite data with geostationary weather satellite; ii) statistical models for intra-hours applications with Probability Density Function (PDF) with a relative Root Mean Square Error (rRMSE) maximum of 22% with a median of 12% [1], with an interesting result for short-term moving average applied to long-terms, but the restriction is the historical data availability, at least 18 years of an specific area [5]; and iii) dynamics models based atmosphere description and radiative transfer models called Numerical Weather Prediction (NWP) and Weather Research and Forecasting (WRF) models, associated to aerosol and clouds absorption with rRMSE of 12% in Winter and 24% for summer season [2].

Another point is the tool used, the last year has considered recurrent neural network for the prediction of the irradiance, however, the active power and the compensation of the DC/AC inverter change the output of the power for two to five minutes with more active power according the reserve and setting point in the PV solar plant; even if the best condition is appropriated (without clouds) The best RMSE and R2 values of 46.1 W/m^2^ and 95.8 % respectively, were obtained from the directional gated recurrent unit (Bi-GRU) model with high values of clear sky, the results are not similar for clouds and irregular radiation; it requires other artificial neural network (ANN) for this prediction [3]. On the other hand, the regression enhanced incremental self-organizing neural network (RE-SOINN) allows to increase the accuracy with a RMSE of 73.94 W/m^2^, compared with the persistence model 103.94 W/m^2^, exponential smoothing 91.46 W/m^2^ and artificial neural network with 90.55 W/m^2^; a clear limitation in the RE-SOINN is the optimal hyperparameter to suit a specific climatic trend; therefore a learning process should be considered in the learning process as Particle Swarm Optimization (PSO) or similar algorithms [4].

Consequently, the motivation of this research article is the comparison and proposal of the three technologies by using the best indicator recommend for the short-term forecasting, with mean absolute error (MAE) [6]; it also enhances the security of grid operation [17]. This comparison will be for the largest PV solar plant in Peru with tracker system, the new contribution is the complementary of these technologies in order to improve the short-term forecasting.

This research article is composed with the four sections. Section 2 develops the methodology, tools, and algorithms, besides section 3 has the results of the case the study with the limitations and contributions, finally, section 4 has the conclusions and future recommendations.

## 2 Methodology and tools

### 2.1 Error rate sensibility

According to the authors [6] and [7], the MAE represents the sensibility and mitigate the signs of the error and evaluates an estimate of the average deviations, according to Eq. (1).

(1) $$MAE=\frac{1}{n}\times \overset{n}{\underset{j=1}{\sum }}\left|y_{j}-y_{j}^{\prime }\right|$$

where:

$y_{j}$: It is the measurement of the actual variable.
$y_{j}^{\prime }$: It is the estimation.
*n*: It is the quantity of the observations.

The MAE of the irradiance represents an average of how many irradiances (W/m^2^) is the estimation away from the true value and the root mean square error (RMSE), it is sensitive to large deviation between forecast and real values [8], in Eq. (2).

(2) $$RMSE=\sqrt{\frac{\sum_{j=1}^{n}\left|y_{j}-y_{j}^{\prime }\right|}{n}}$$

And the mean absolute percentage error (MAPE), associated to the accurate of the forecast system in Eq. (3), as a mean absolute percentage error.

(3) $$MAPE=\frac{\sqrt{\sum_{j=1}^{n}\frac{\left|y_{j}-y_{j}^{\prime }\right|}{\left|y_{j}\right|}}}{n}$$

with *n* is the number of times of the iteration, $y_{j}$ is the actual value, $y_{j}^{\prime }$ is the forecast value.

### 2.2 Data collection

A comparison of the last 36 months of the active power, irradiance, clear index, for the pyranometer the global horizontal irradiance, tilted horizontal irradiance, irradiance from satellite data and wind velocity; the analysis considered the MAE in all the period.

### 2.3 Mathematical description

The first approach obtained is the Ridge regression with satellite data, it is centered with according the multi-classification problem, it provides according to the training set in Eq. (4) and the objective function indicated in Eq. (5).

(4) $${\left\{z_{j},x_{j}\right\}}_{j=1}^{n}$$

(5) $$\begin{array}{l} min\frac{1}{2}{\left\|W\right\|}_{F}^{2}+\frac{\theta \sum_{j=1}^{n}{\left\|\rho_{j}\right\|}^{2}}{2}\\ s.t.\;W^{T}\sigma \left(z_{j}\right)=l_{j}-\rho_{j} \end{array}$$

where:

$z_{j}$ ε R^C^; it is the one-hot label of $z_{j}$ and C is the categories.
σ denotes the kernel function.

**Figure 1:**
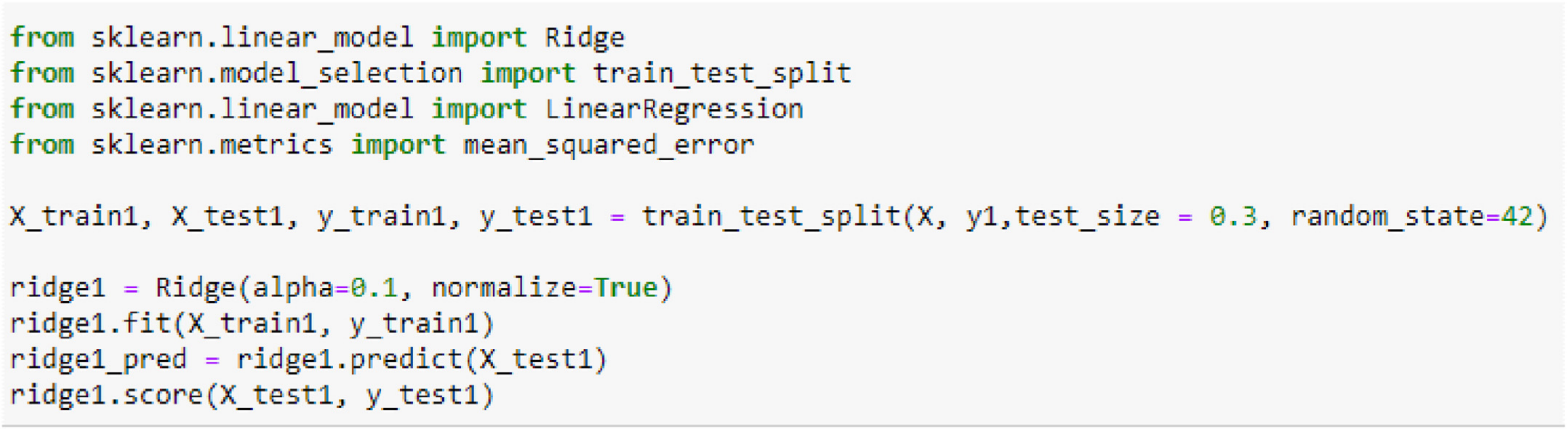
Ridge algorithm for the training sections.

In Figure 1 the ridge regression has a particular form of constraints with Eq. (6) and penalized sum of squares in Eq. (7).

(6) $$\beta =\frac{X^{\prime }Y}{\left(X^{\prime }X+\lambda I_{p}\right)}$$

(7) $$\overset{n}{\underset{j=1}{\sum }}{\left(x_{j}-\overset{p}{\underset{i=1}{\sum }}y_{j}\beta \right)}^{2}+\lambda \overset{p}{\underset{i=1}{\sum }}\beta^{2}$$

where:

β is the with the estimator and the λ is the small constant value to the diagonal entries of the matrix and p is the constraint in ridge regression.

**Figure 2:**
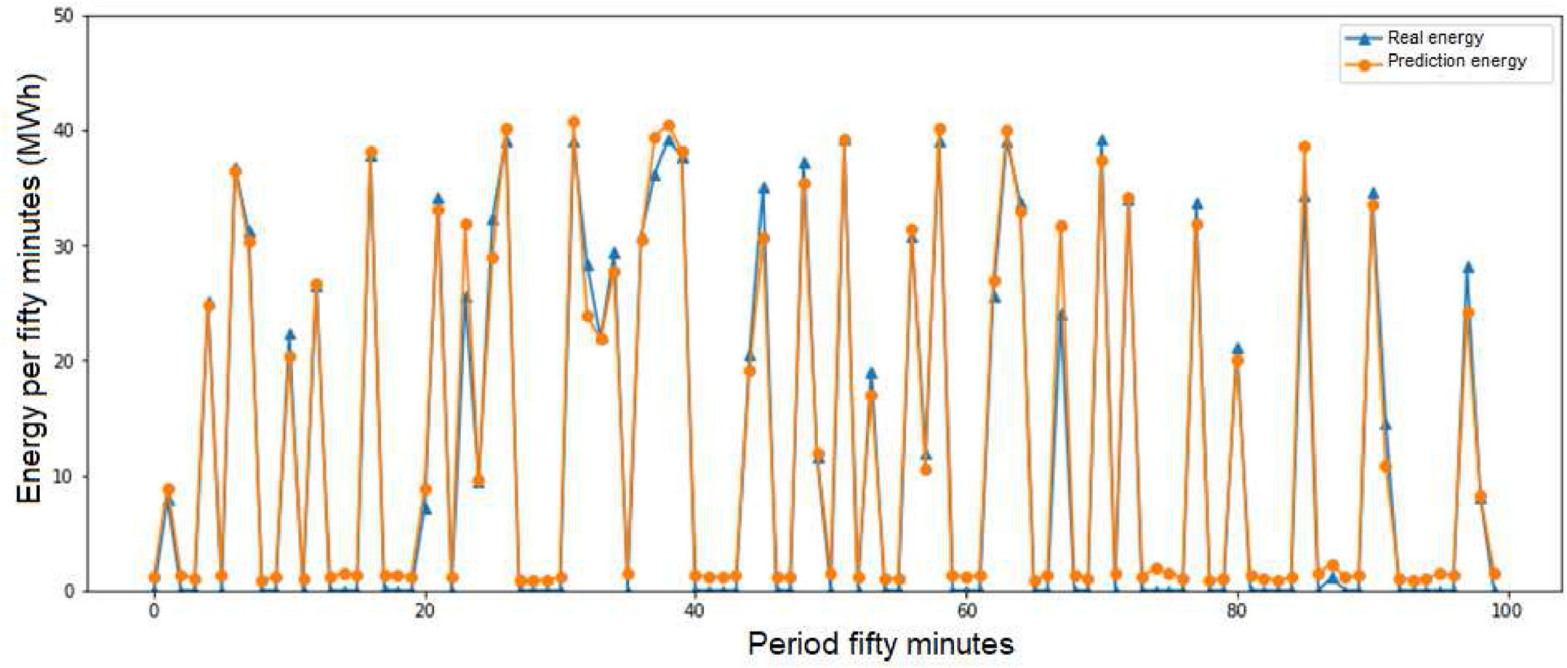
Satellite data training set.

The ridge regression score is 97.0618% and the root mean squared error (RMSE) is 2.6335 and the MAE is 1.7876; according to Figure 2, for clouds conditions.

Designing a global tilted irradiance (GTI) forecasting is a complicate study, the use of the specific spatial—temporal context is important for each PV solar plant [8]. The PV solar plant considers trackers, the global tilted irradiance evaluates in forty-one pyranometers, the ridge analysis considers timestamp, months, global horizontal irradiance, and ambient temperature; the satellite data is designed with a small region of 2 km × 2 km with the irradiance analysis of the satellite, clear index in Eq. (8), GTI, GHI and global diffuse irradiance (GDI) [9]. The Long Short-Term Memory (LSTM) is used for the feedback in the ridge regression, associated to the GTI [8].

(8) $$ClearIndex=\frac{GDI}{GHI}$$

The dynamics of the LSTM model is associated to Eq. (9) and Eq. (10), [10].

(9) $$h_{t}=\sigma \left(Ux_{t}+v_{h}h_{t-1}\right)$$

(10) $$y_{t}=W\times h_{t}$$

where:

$x_{t}$: It is the sequential input from the temperature, irradiance, wind velocity.
$h_{t-1}$: It is the internal short-term memory with a weight called W for the output.
σ: It is the sigmoid activation function.
$y_{t}:$ It is the prediction model associated to irradiance GHI, GTI, wind velocity, ambient temperature.

**Figure 3:**
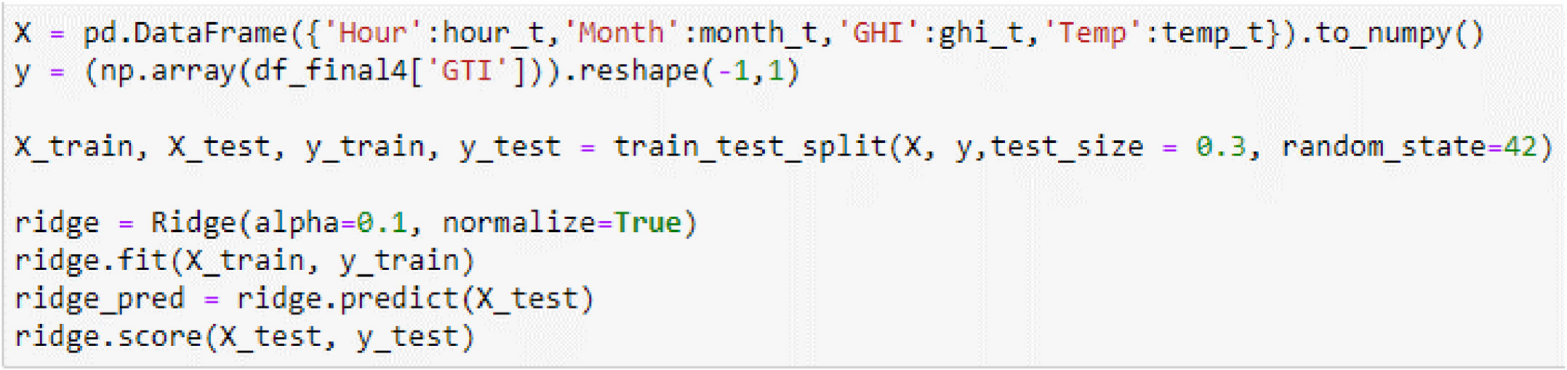
Global tilted irradiance (GTI).

With the RNN model, the main restriction is the “shortcoming of vanishing gradient that prevents them from updating the weights during training process, according to previous time lags” [10]; it is solved with the LSTM and ridge models (see Figure 3).

**Figure 4:**
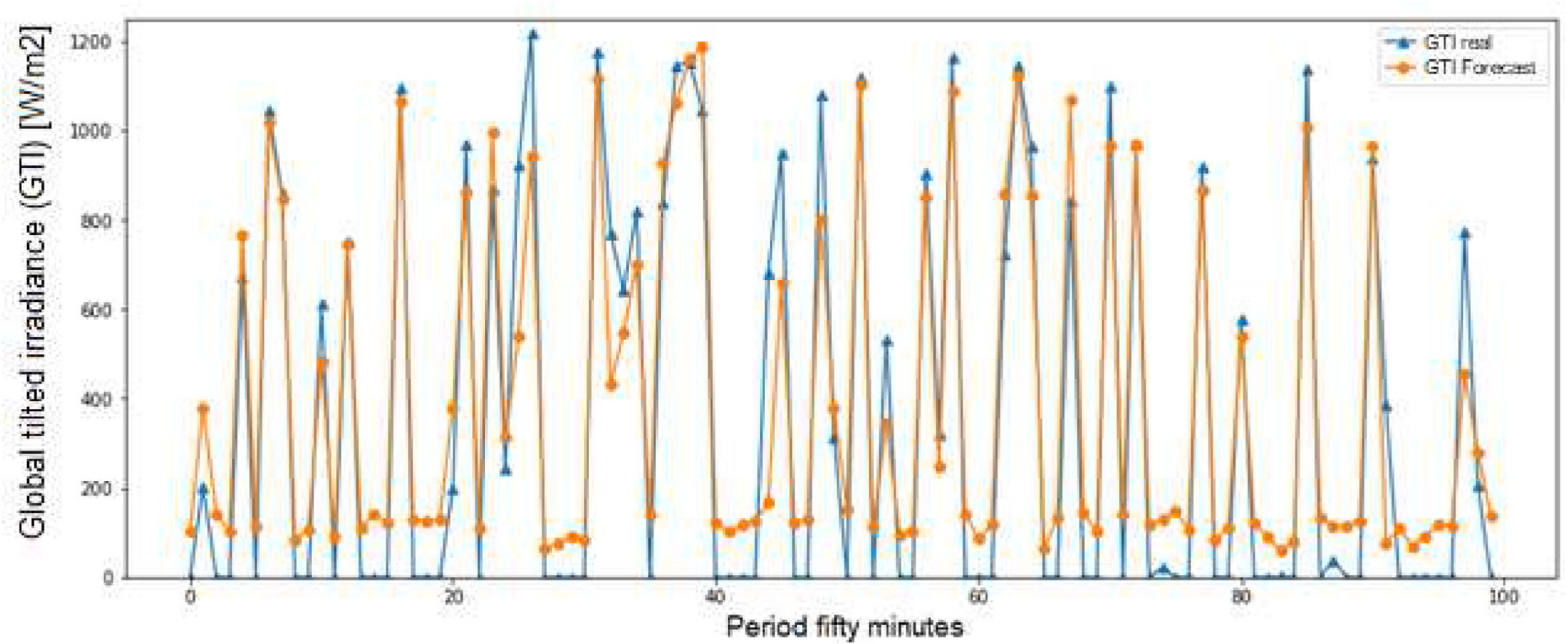
Training evaluation in the global tilted irradiance.

For the GTI evaluation, the ridge score is 78.3099%, the RMSE is 204.7590 and the MAE is 142.8901, according Figure 4 for 1 day.

Consequently, the active power prediction has the ridge score 97.1061%, the RMSQ is 10.5343 MW and MAE 7.1506 MW.

**Figure 5:**
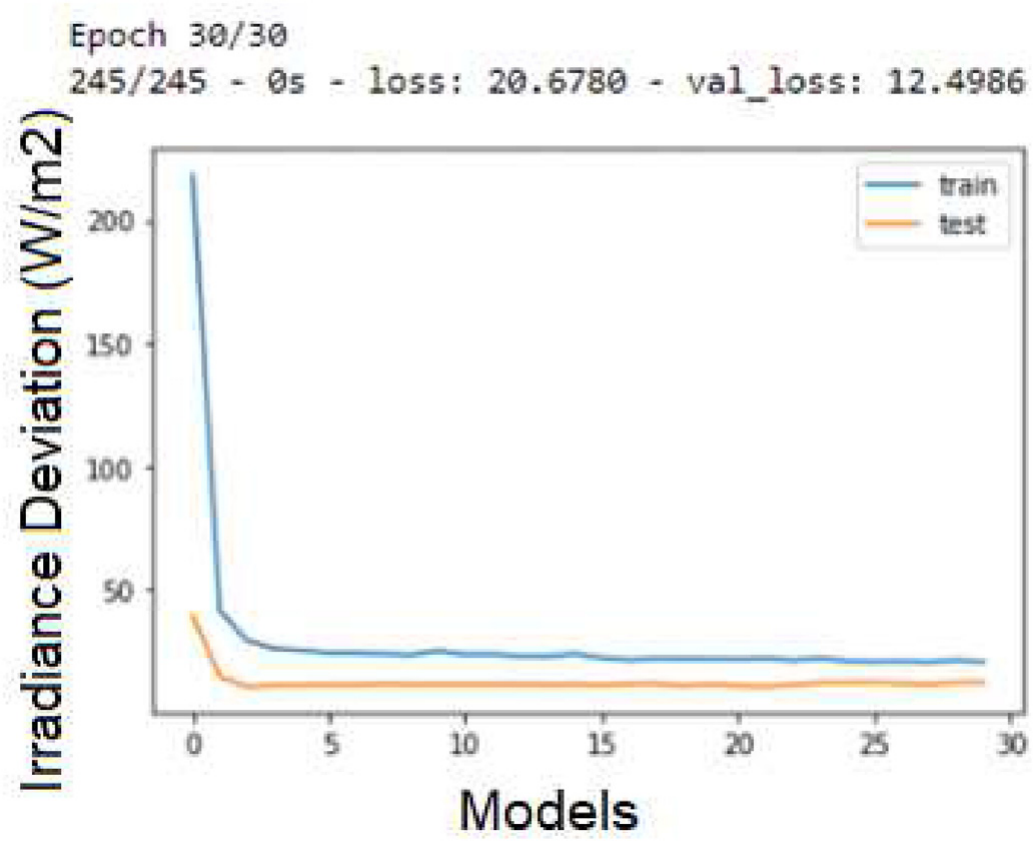
LSTM for temperature prediction.

**Figure 6:**
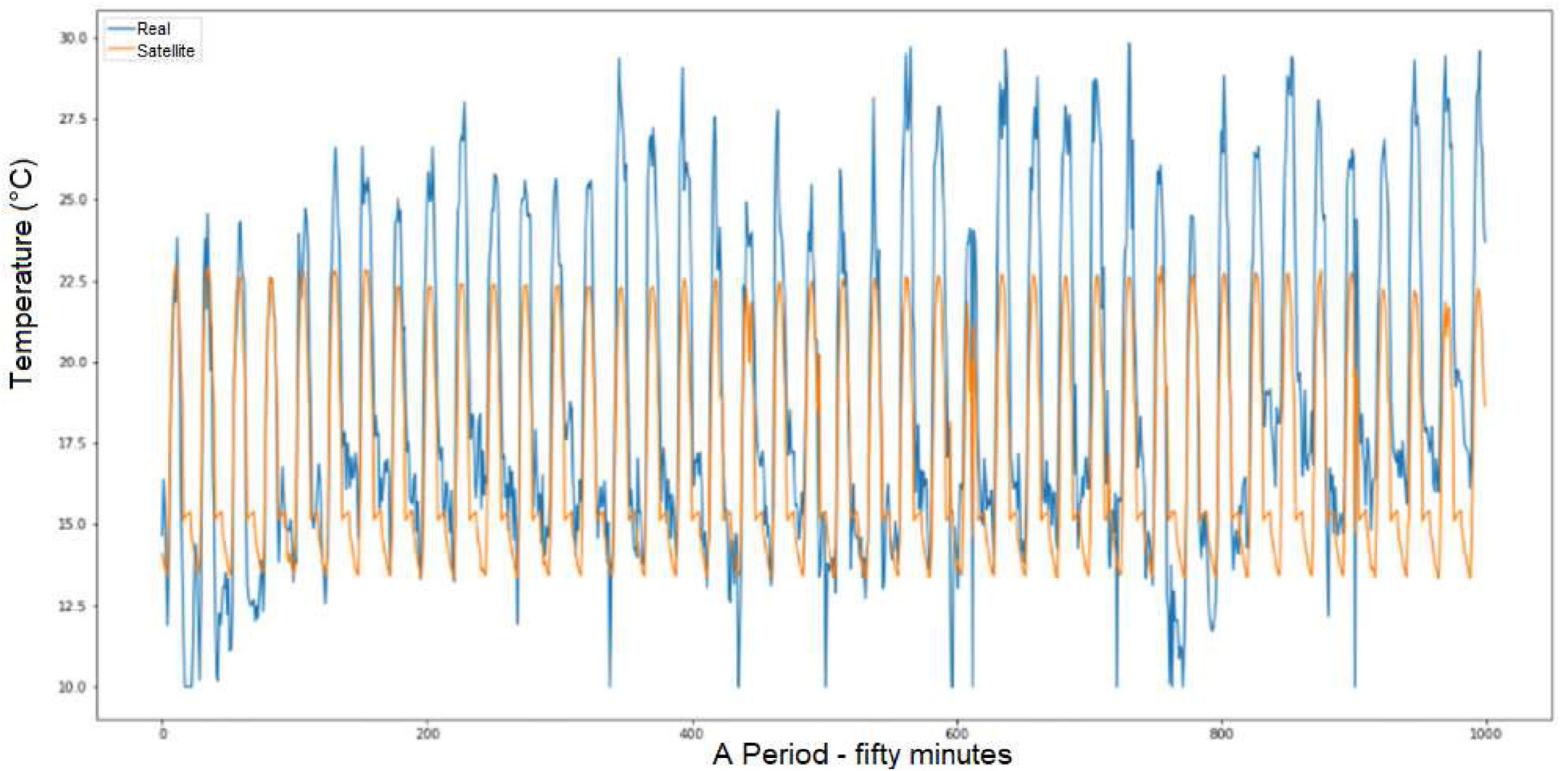
Temperature prediction with LSTM-CNN.

About the improvement with deep hybrid LSTM–CNN model, which integrates LSTM with convolutional neural network (CNN) to model spatial-temporal features for short-term for the evaluation of the temperature and prediction in Figure 5 and the results in Figure 6. It represents a highly stable condition of the season variation and sky condition (clouds over the panels) with 30 epochs; the RMSE is 3.535 and MAE 2.699 for a prediction of ten days.

**Figure 7:**
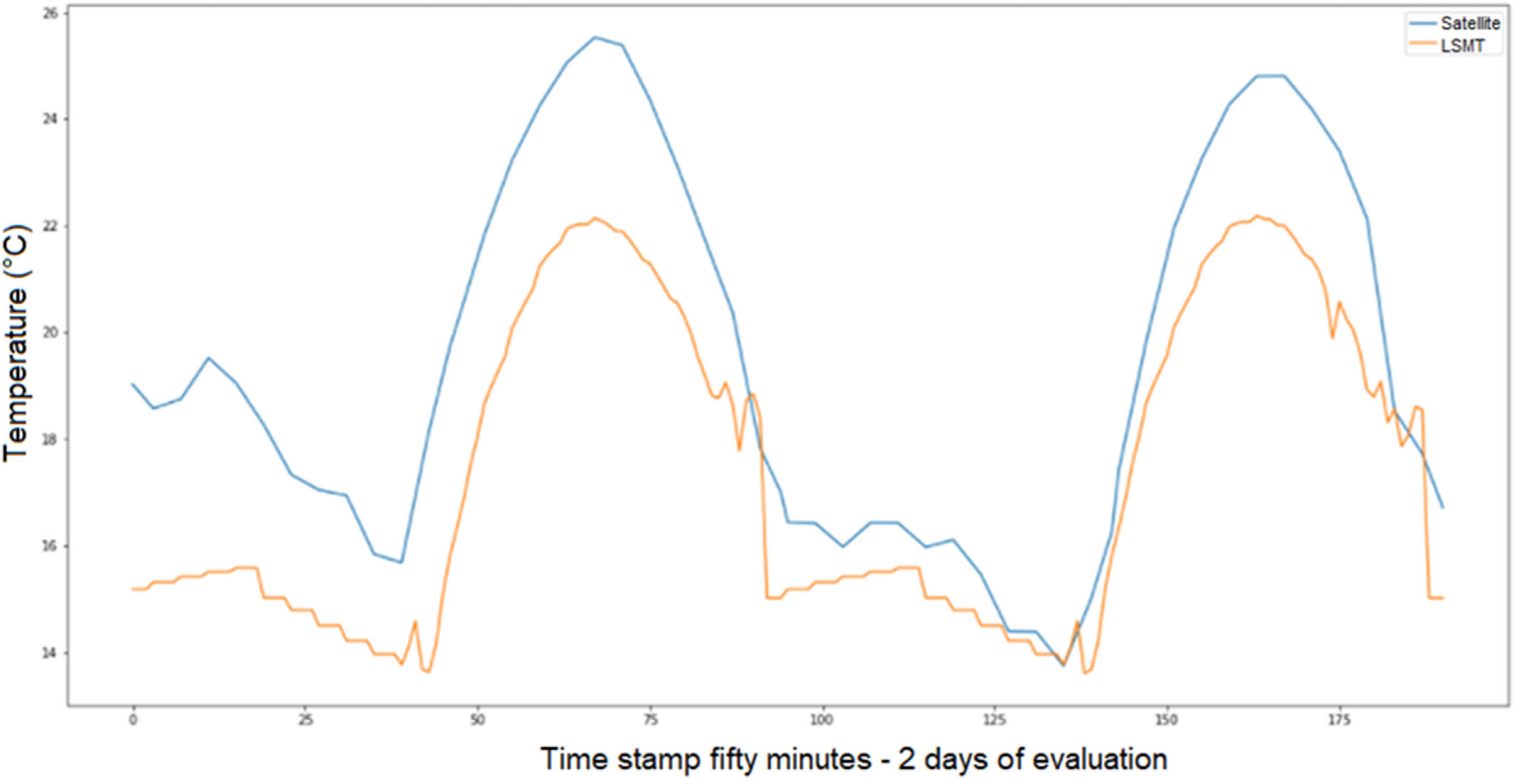
Ambient temperature prediction with LSTM-CNN.

Besides, the satellite temperature and the prediction model from the satellite and the LSTM-CNN model with a RMSE 2.439 and the MAE 2.5065, in Figure 7.

One of the challenges in tropical zones is the ambient temperature, from the satellite data, it requires a LSTM CNN for the prediction; the algorithms use the parameters according to the Algorithm 1: The neural network used is the Neural Prophet. A clear difference between the NeuralProphet and Prophet model is AR-Net. Therefore, if the scope does not include the AR-Net to NeuralProphet, then it expects that “Prophet and NeuralProphet would learn the same parameters and get the same performance” [11].

The NeuralProphet model has the following dynamic in Eq. (11).

(11) $$y_{t}=g_{t}+s_{t}+h_{t}+\omega_{t}$$

(12) $$g_{t}=\left(k+{a_{t}}^{T}\times \mu \right)t\left(m+{a_{t}}^{T}\times \gamma \right)$$

where:

$g_{t}$: It is the trend function for non-period changes in the source.
$s_{t}$: It is the representation of the season, in Eq. (11).
$h_{t}:$ It is the irregular condition from holidays and sudden changes for Pandemic and others.
$\omega_{t}$: It is the error term in the model for each verification.
*k*: It is the growth rate.
*m*: It is offset.
μ: It is the trend change points.
${a_{t}}^{T}\times \mu$: it is the continuous function.

**Figure 8:**
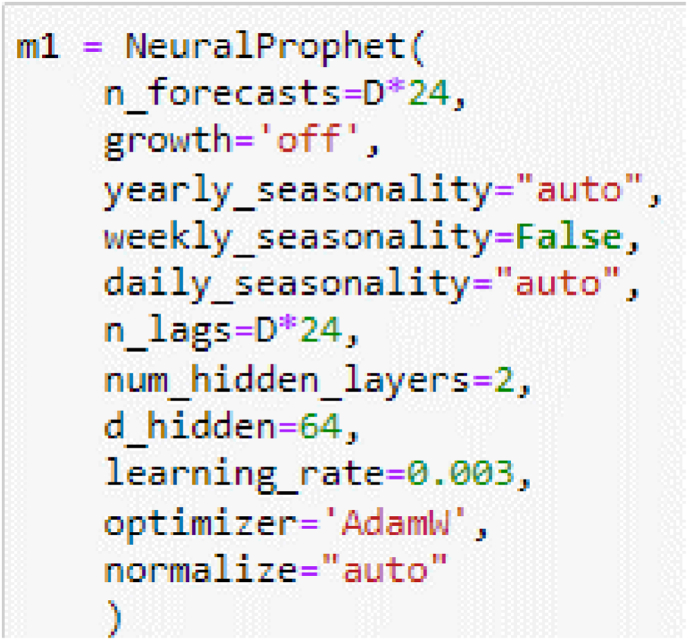
Declaration of the neural prophet for the dimension and hidden layers.

In Figure 8, the algorithm 1 is described according to Eqs. (11) and (12), it considers seasonality and a daily evaluation with at least seven days for the AR-NET and 2 hidden layers with 64 dimensions of hidden layers.

### 2.4 Methodology proposed

**Figure 9:**
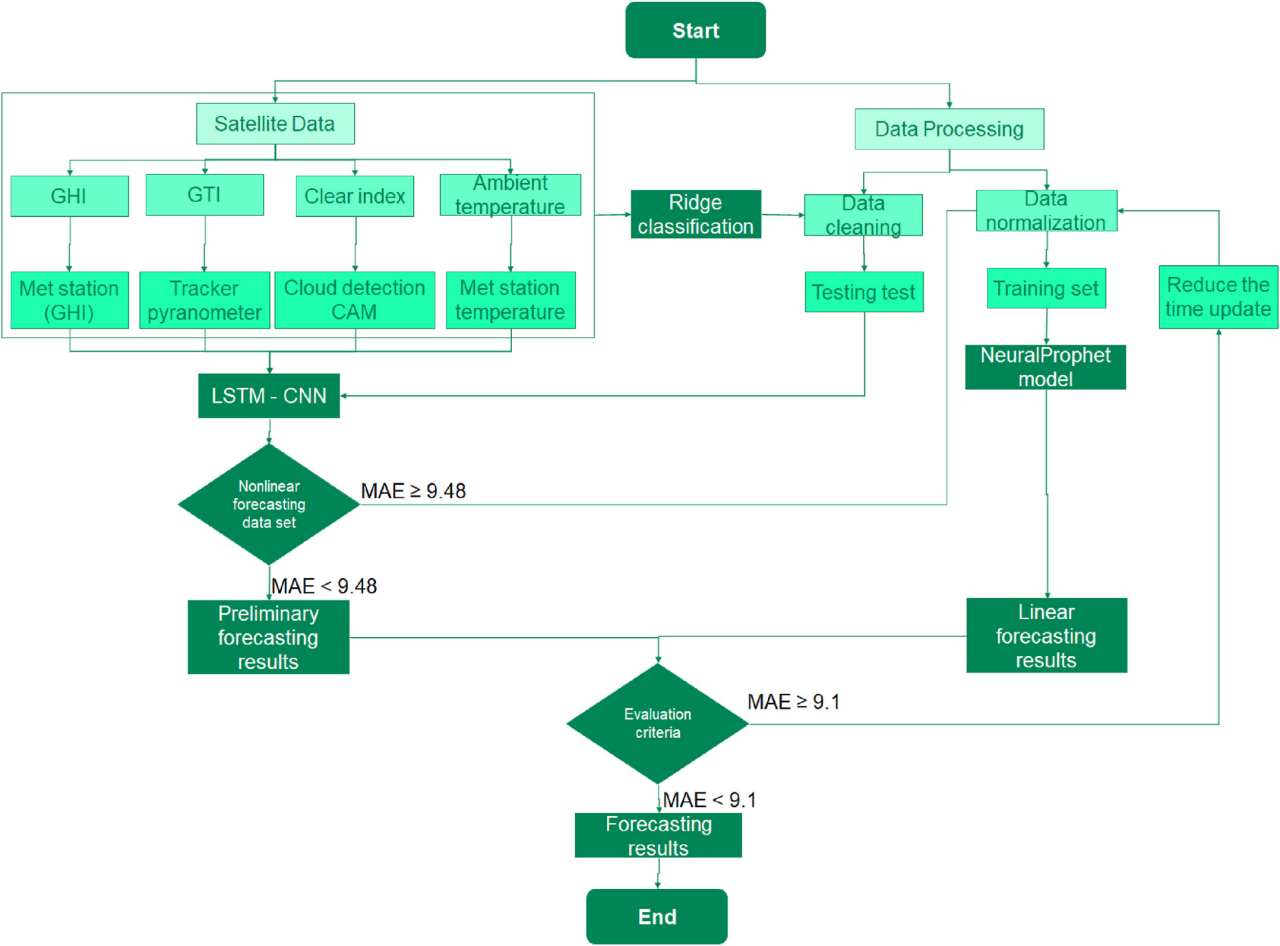
Methodology proposed for forecasting.

Usually, the forecasting techniques based on historical data is composed by four approaches: i) The persistence method, ii) the statistical, iii) the machine learning process, and iv) the hybrid techniques [17]; therefore, this methodology combines the machine learning with the hybrid techniques. The main contribution of this research article is the proposal of a hybrid model, it combines the NeuralProphet advantages with LSTM-CNN; due to high accuracy in seasonal time series by an automatic calibration of the satellite data with meteorological station with the ridge classification process. Compared with traditional process, the benefits allow to improve the MAE, RSME. The steps are four in Figure 9, as follows:

Step 1: The Data processing is divided in two processes as Data cleaning and data normalization; associated to the testing set and training set.
Step 2: The evaluation of the satellite data and meteorological (MET) station, in this case the GHI is used with the calibration of the horizontal pyranometer installed in the MET station, GTI data is used with the pyranometers installed in the trackers; the clear index is improved with the cloud detection CAM installed in the PV solar plant and the ambient temperature, calibrated with the MET station temperature; in this case, the ridge classification allows to improve the data cleaning and testing set for the step 1.
Step 3: The NeuralProphet model is a curve fitting of time series data. In parallel, the adaptability to seasonality and trend change point is important, with an entropy evaluation, due to linear forecasting result and the Long Short-Term Memory with convolutional neural network for the preliminary forecasting result in linear and nonlinear results.
Step 4: The evaluation criteria uses the MAE and RSME, in order to improve the last result, if the values are higher than objective, then the data processing is improved with a new ridge classification with the satellite data, for the normalization and training set.

## 3 Case study

**Figure 10:**
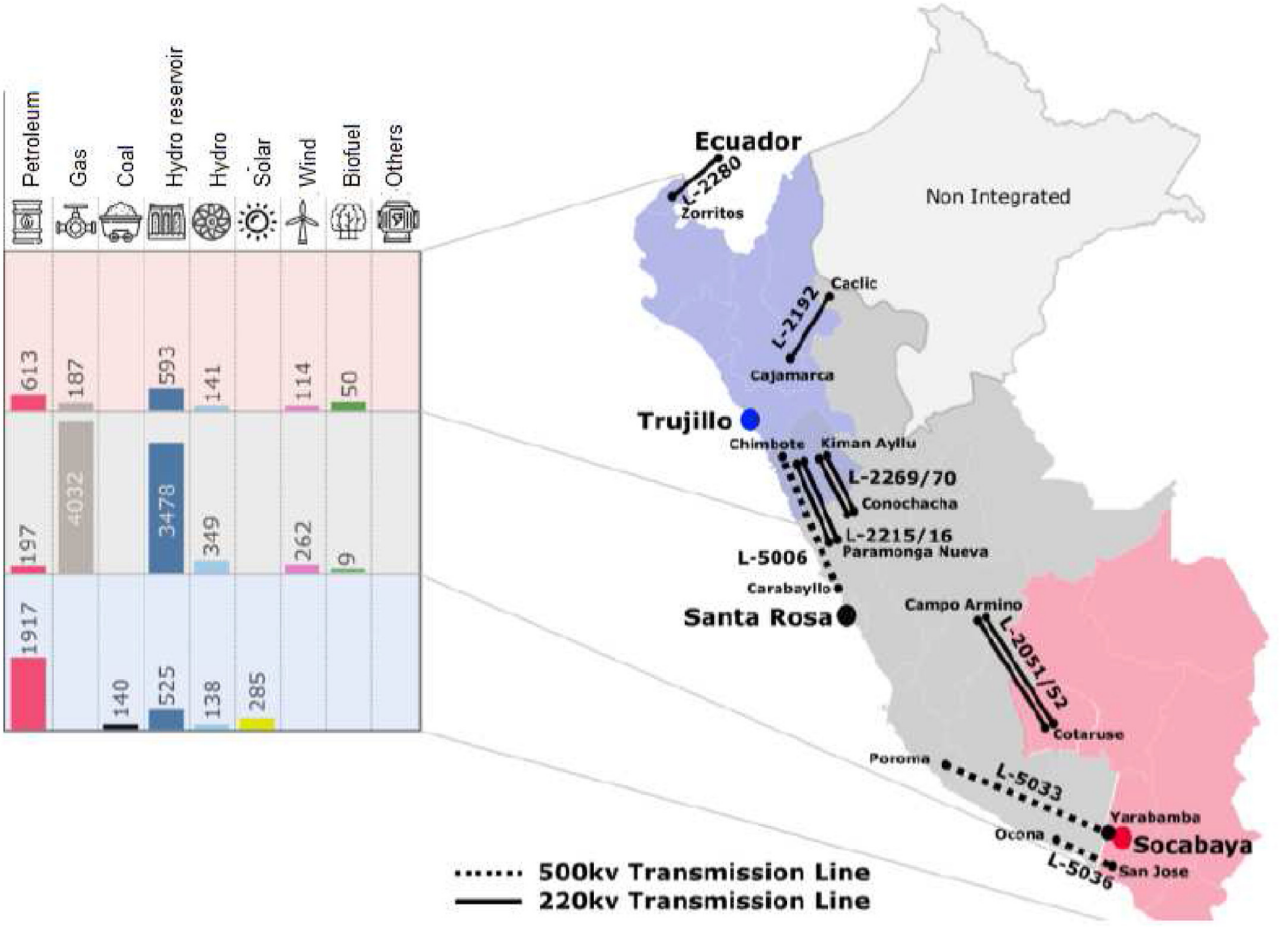
Case study selection.

**Figure 11:**
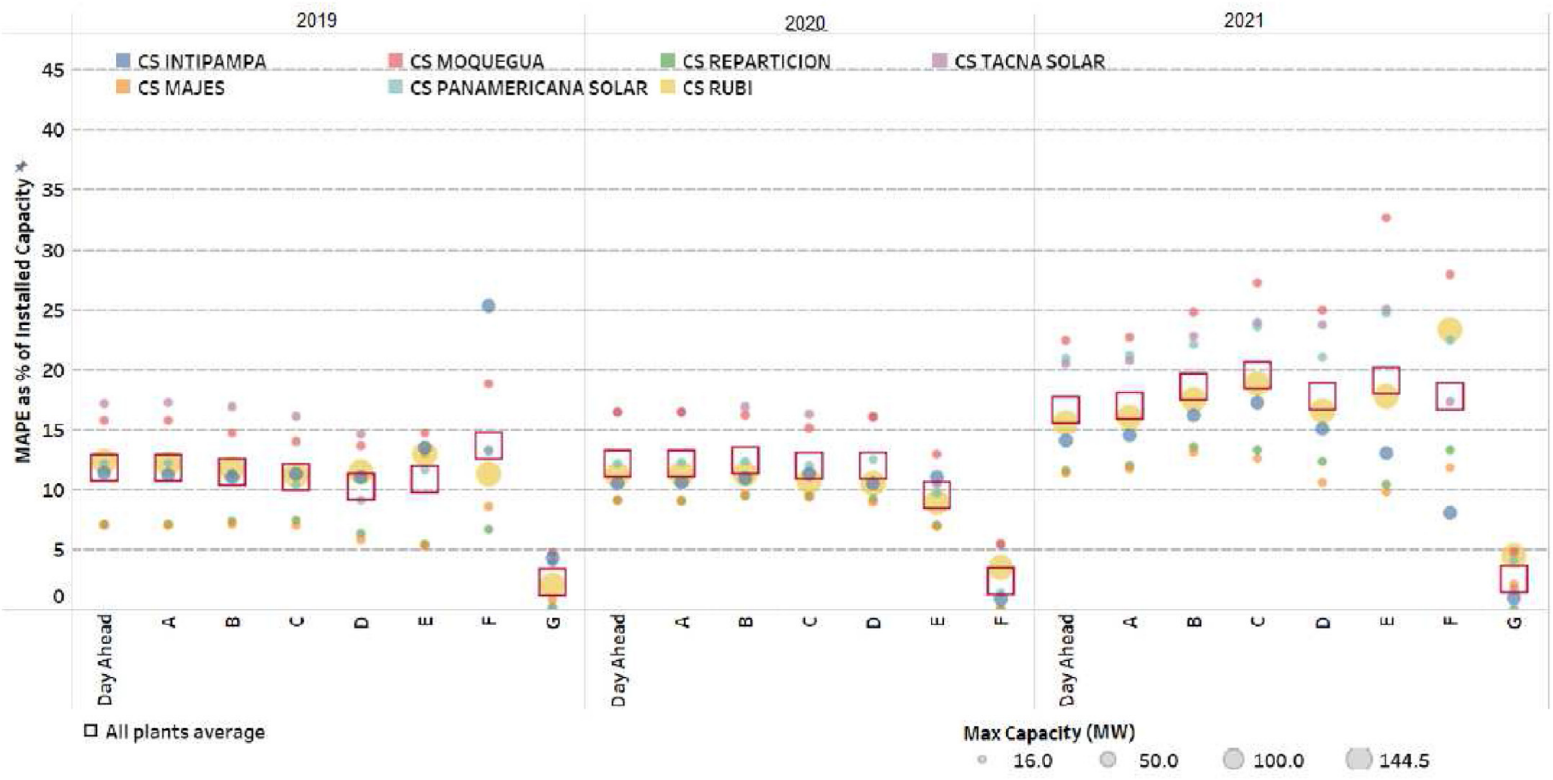
MAPE for day ahead forecasting.

For the case study, the country selected was Peru, it has 285MW of PV solar plants centered in the south of Peru with six PV solar plants, in Figures 10 and 11; therefore, the MAPE as a percentage of the installed capacity, for Peruvian grid, the solar plants, for the same period (January 2019–May 2020), with respect real generation and the forecasts. The time block between 05:30 and 18:30 is considered, since in the rest of the hours the solar generation in the grid is null. Figure 11 shows that the average deviation, with respect to the forecast of the day before, has been between 10% and 15% between the years 2019 and 2020, reaching deviations of 20% in the year 2021. No improvement is observed substantial in the performance of intraday rescheduling forecasts, relative to the prior day's scheduling.

The information available for the case study is included in this research article, as supplementary materials:

• AppV1 .ipynb
• Pot_15m .xlsx

In Peruvian PV solar plants, the average MAPE of 12.1% in a clear index of 83.18% with a minimum of MAPE 3% with clear index of 99.1% and a maximum of 26% with a clear index of 58.12%. Therefore, the KPI indicators: MAE, RMSE and MAPE is 8.58 MW, 12.86 MW and 5.93% respectively.

The case study was evaluated in the biggest PV solar plant in Peru, with 179.8 MWdc or 144.5 MWac, with 0.56 million of panels; it is the 63% of the total PV solar plant in Peru.

### 3.1 Short-terms forecasting

In residential PV system of 669.624 W uses a genetic algorithm-based support vector machine (GASVM) model; the results are important in low scale for short-term power forecasting by a difference of the RMSE value and 98.7648% of the MAPE error [16]; however, the challenge is to implement a short-term forecasting algorithm for large PV solar plants.

**Figure 12:**
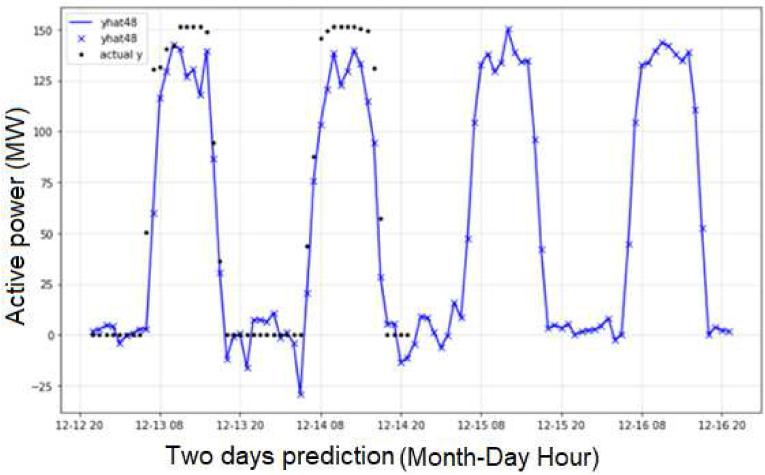
Preliminary results with NeuralProphet evaluation for two days.

**Figure 13:**
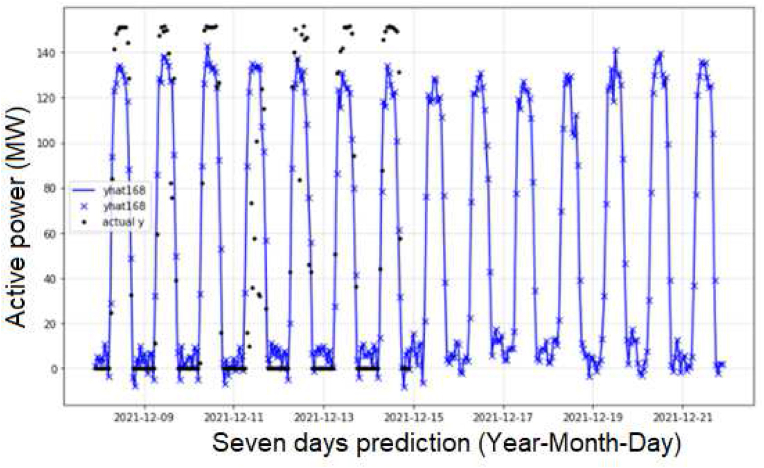
Results with NeuralProphet and LSTM-CNN and ridge classification.

**Figure 14:**
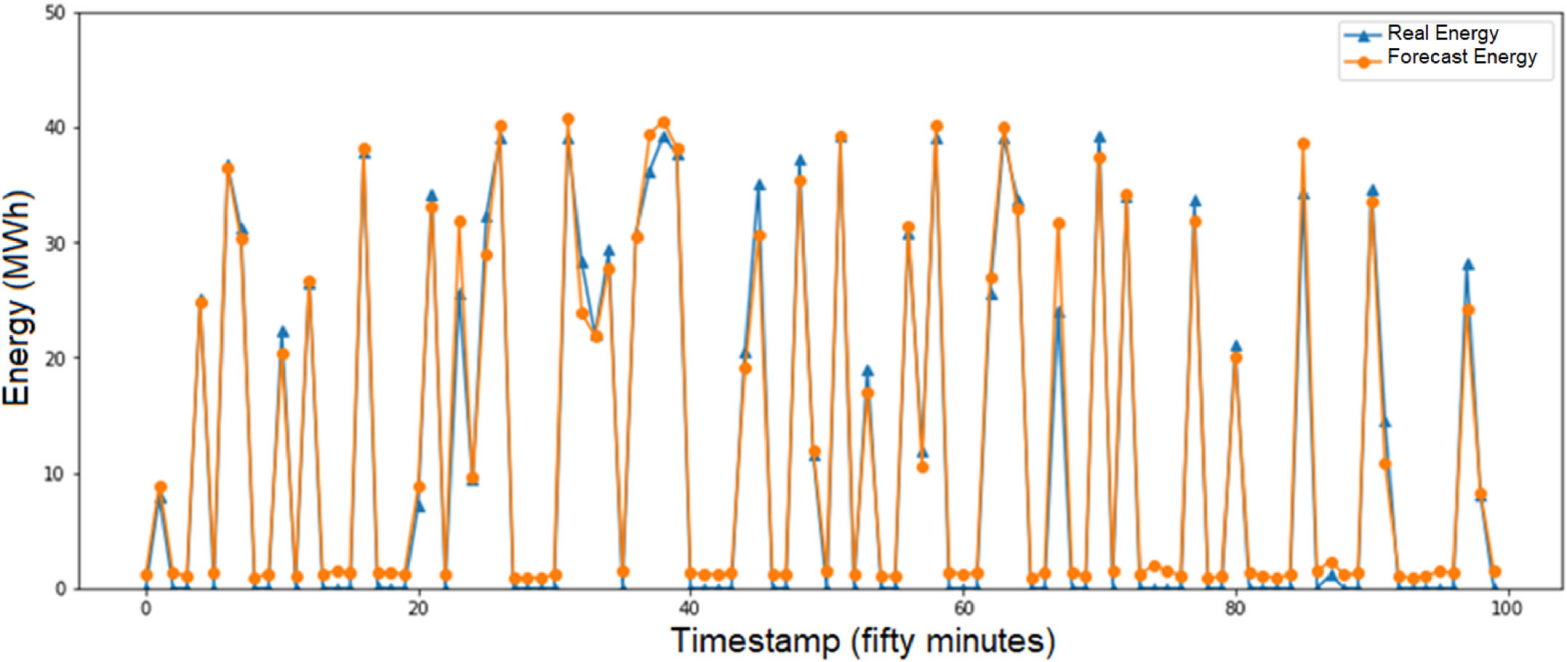
Energy prediction with the model.

**Figure 15:**
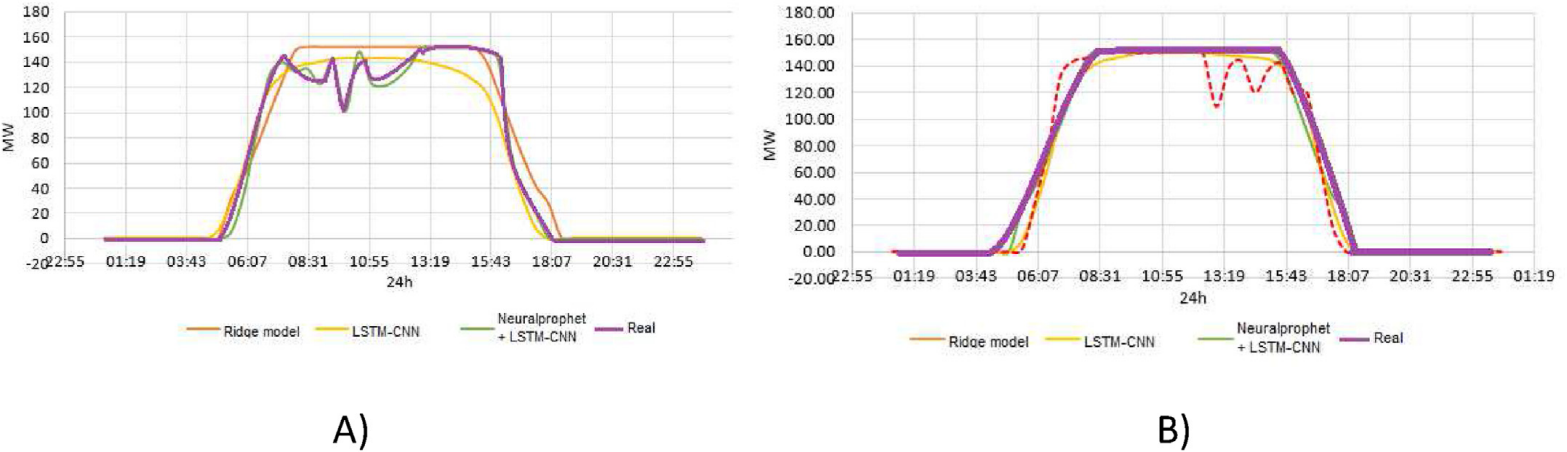
Evaluation of the models. A) Evaluation with clouds over the PV solar plant. B) Evaluation without clouds.

About the case study proposed, with the evaluation of two day in Figure 12, and seven days in Figure 13 for the prediction of the active power in MW, besides, Figure 14 shows the evaluation of the energy generated per day, and the results of the training process for one day in the period of fifty minutes and the evaluation of Figure 15. In Figure 15 A, the evaluation with clouds has a MAPE of 5.01% and 8.14 MW and Figure 15 B has a MAPE of 2.2% and 3.62 MW.

The evaluation for 2021 during November and December 2021, the information indicated in Table 1.

**Table 1:** Result of the model proposed.

Description	Smooth 1 Loss (per unit)	MAE (MW)	RMSE (MW)	MAPE (%)
Prediction of one day (A day ahead (update))	0.007338	9.417517	17.989578	0.06517313
Prediction of two days (A day ahead (update))	0.008063	9.910001	18.855035	0.06858132
Prediction one day without Ridge model (A day ahead (update))	0.008685	10.763111	19.617117	0.0744852
Prediction of two days without Ridge model (A day ahead (update))	0.009509	11.484186	20.529688	0.07947534
New model Prediction one day (twelve-hour update)	0.006212	9.112122	15.122432	0.06305967
New model Prediction one day (six-hour update)	0.0059223	8.9212885	13.2312232	0.06173902
New model Prediction one day (three-hour update)	0.0051129	8.5757739	12.8636311	0.05934792

The evaluation of the.

## 4 Discussion

In Peruvian PV solar plants, the average MAPE of 12.1% and the evaluation each three-hours with the proposed model, the MAPE is 5.93%. In Table 2, about the evaluation with new original methodologies, the original dataset is used to evaluate with LSTM with ARIMA, Genetic algorithms (GA), Gaussian approach (G), Prophet and Support Vector Machine (SVM); the selection include two aspects:

**Table 2:** Comparation for the same dataset and several technologies in large scale PV solar plants.

Description	Year	MAE (MW)	RMSE (MW)	MAPE (%)
SARIMA and SVR considering Gaussian White Noise [13]	2019	11.17362	17.70117	8.12%
Evaluation of six companies in Peru	2021	17.54423	26.46128	12.14%
ARIMA-LSTM	2021	15.27128	23.03308	10.57%
LSTM with neural network and genetic algorithms [12]	2021	11.72638	17.7428	8.14%
Prophet with Support Vector Machine [14]	2021	11.19726	17.82691	8.81%
New model Prediction one day (three-hour update) NeuralProphet & LSTM-CNN with Ridge regression (Historical & Satellite data)	2022	8.575774	12.86363	5.93%

First of all, the original data could be obtained directly from the satellite and the PV solar plant.

The comparison between the computational time is evaluated and optimized in Python 3.8.3, the system is: 64 bit Quad-Core Intel Core i5 GPU @ 2 GHz, 3733 MHz and 32Gb ram installed.

**Figure 16:**
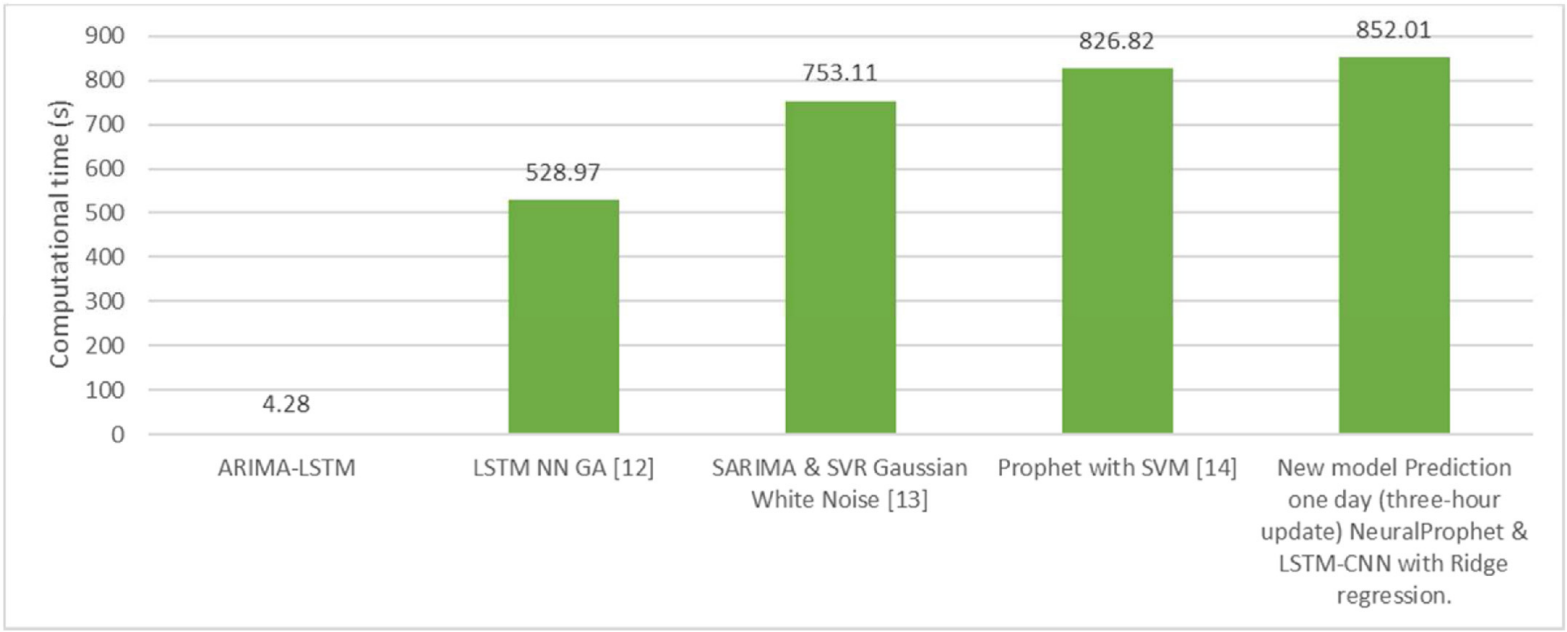
Computational time compassion between technologies.

The deep model LSTM, Ridge, SVM, Genetic algorithm, Prophet and NeuralProphet is implemented with free libraries in Anaconda. The execution and training process for the same computer, is 14.2 min with the new model prediction, more time compared with the ARIMA, however, the complexities of the LSTM and support vector machine and Prophet are the dimensions, Kernel function and linear time complexity; according to Figure 16.

Finally, one of the most difficult aspects is the prediction of the clouds over the PV solar plant, it could add the “ability to improve short-term prediction by the new regularization-based techniques demonstrates” [15] and according to the resource availability and external influence as the cloudy condition.

**Figure 17:**
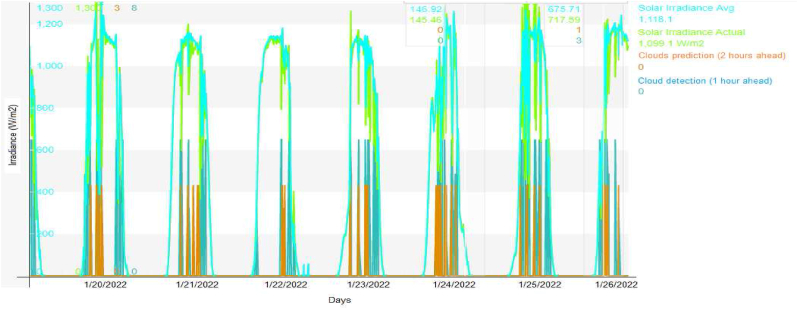
Results of the clouds detection and pyranometer evaluation.

**Figure 18:**
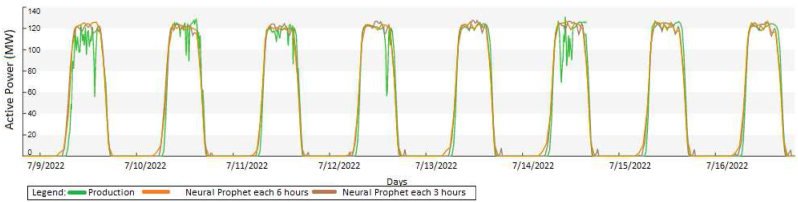
Evaluation of the active power with the irradiance.

In this case, Figure 17 evaluates the results of the prediction 2 h ahead and 1 h ahead during January 2022, according to the recommendation of the authors in [18]. Besides, in Figure 18 evaluates the period in July 2022 with three to six hours ahead according to the recommendation of the authors in [18], for active power as follows:

• Irradiance: Two hours ahead has an accuracy of 83.4% of the cloud’s prediction over the PV solar plant.
• Irradiance: One hour ahead has an accuracy of 90.9% of the cloud’s prediction over the PV solar plant.
• Active power: Each six hours ahead; it has an accuracy of 82.6% of the cloud’s prediction over the PV solar plant.
• Active power: Each three hours ahead; it has an accuracy of 89.1% of the cloud’s prediction over the PV solar plant.

## 5 Conclusion

In this research article a new model with satellite and PV solar plant data improves the input for the processing, the main contribution is the accuracy in the model; and it combines Ridge classification for the evaluation of the satellite and MET station, besides, the LSTM-CNN and NeuralProphet improves the linear and non-linear challenge in the seasonality analysis and characteristics. The NeuralProphet allows to improve the evaluation of the fluctuations. The New model reduces the currently prediction of six companies in Peru with a MAPE 12.14%–5.93%; even compared with the literature and currently models as ARIMA-LSTM with 10.57%, LSTM with NN and G, SARIMA and SVM considering Gaussian White Noise with 8.14% and Prophet with SVM with 8.81%; therefore, the evaluation improves and contribute with higher accuracy, although the computational time is increasing in the training process; besides one the challenges are the clouds prediction, in this case, the best result is the cloud’s prediction one hour ahead with the model proposed in this article.

The contribution is the combination with other models for the data processing, in this case the incorporation of the satellite data with geostationary weather and the statistical models with the evaluation of the density function and dynamic models for the seasonality analysis; in a brief summary, the combination of the three perspectives allows to reduce the noise and to reduce the error; with a strongest and robust method. Finally, the limitation of the study is the quality of the clouds detection with the satellite, it is usually 4.5% [19], besides, the computation capacity with data more than 3 years, with 42 TILTED pyranometers, 3 GHI with the software Python 3.8.3, the system is: 64 bit Quad-Core Intel Core i5 GPU @ 2 GHz, 3733 MHz and 32Gb ram installed; it has 2.1 h of computation time for the training process.

## Declarations

### Author contribution statement

Ricardo Manuel Arias Velásquez: Conceived and designed the experiments; Performed the experiments; Analyzed and interpreted the data; Contributed reagents, materials, analysis tools or data; Wrote the paper.

### Funding statement

This research did not receive any specific grant from funding agencies in the public, commercial, or not-for-profit sectors.

### Data availability statement

The information is available attached as Supplementary material and CSV files.

### Declaration of interests statement

The authors declare no conflict of interest.

### Additional information

Supplementary content related to this article has been published online at http://doi.org/10.1016/j.heliyon.2022.e10639.

## Appendix A Supplementary data

AppV1.ipynb AppV1.ipynb

Pot_15m Pot_15m
